# Fatigue, Pro-Social Attitude and Quality of Life as Predictors of Empathy in Medical and Social-Oriented Students

**DOI:** 10.3390/ijerph192315853

**Published:** 2022-11-28

**Authors:** Agata Zdun-Ryżewska, Krzysztof Sobczak, Agata Rudnik

**Affiliations:** 1 Department of Quality of Life Research, Faculty of Health Sciences, Medical University of Gdańsk, 80-210 Gdańsk, Poland; 2 Department of Sociology of Medicine and Social Pathology, Faculty of Health Sciences, Medical University of Gdansk, 80-210 Gdańsk, Poland; 3 Institute of Psychology, University of Gdansk, 80-309 Gdańsk, Poland

**Keywords:** empathy, fatigue, compassion fatigue, students

## Abstract

Empathy is significant in professions that require establishing proper contact as a condition for providing help. Identifying factors related to empathy is important for understanding how to teach empathic behavior. The main goal of this study was to find variables related to empathy in a group of students from two universities: medical and social oriented (N = 1701). The study group consisted of female (81%) and male (19%) participants, aged between 18–20 (37%), 21–23 (49%), or 24 years and above (14%). A self-designed questionnaire was used to collect socio-demographical information, with additional questions (social self-esteem, prosocial attitude, subjective quality of life). Empathy was measured with the EQ-40, fatigue with CHFQ-PL, and stress with PSS-10. The results showed a statistically significant regression model for empathy. A high quality of life and having feelings of pleasure when helping other people allows to predict a high level of empathy, especially among females. Higher levels of fatigue and social self-esteem, the latter of which is measured here by the belief that you are more important than others, predicts lower empathy. There were no differences between students from two different kinds of universities when taking into consideration stress levels, subjective quality of life, and prosocial attitude. However, students from the medical university were more exhausted and more convinced that their value was greater than others, as compared to the students studying social sciences. When teaching empathic behavior, it is beneficial to attempt to maintain or restore students’ well-being and reduce fatigue and to teach how to achieve such effects in the future. Learning the balance between compassion, willingness to help, and self-compassion also seems to be important.

## 1. Introduction

When Edward Titchener coined the word empathy, related it to an earlier term, “Einfuhlung”, and defined it as “the process of humanizing objects, of reading or feeling ourselves into them” [1] in the early twentieth century, a new trend of research began. The study of empathy remains popular today due to the importance and role of the concept within any profession in which there is contact with another person. In his patient-centered therapy, Carl R. Rogers mentioned empathy, along with warm acceptance and authenticity, as crucial in establishing a proper therapeutic alliance in every helping relationship. According to Rogers, empathy should influence not only the way that professionals communicate but also their entire attitude towards clients or patients. Being in contact with someone, especially someone who suffers from stress or is prone to feeling vulnerable or anxious, requires an empathic understanding of a person’s subjective perspective [2]. Such an attitude seems to be of particular importance in medical and social professions, from health care providers (which includes doctors of various specializations, nurses, midwives, and physiotherapists) to psychologists, psychotherapists, educators, and teachers. Although the specific goals of each of these professions are different, the general objectives can only be achieved through the fulfillment of the basic needs of a patient, student, or client, and the ability to establish healthy relationships even in highly demanding surroundings [3]. These basic principles influenced the development of two popular research streams. The first topic investigates the relationship between empathy and burnout, assessing the importance of maintaining an empathetic working style among professionals and preventing work burnout [4,5,6,7,8,9]. The second popular topic of scientific investigations concerns teaching and learning empathic behaviors such as self-awareness, good listening skills, non-judgmental communication, and using empathy in the process of understanding another person to prepare future graduates to adequately perform their work-related tasks [10,11,12,13]. 

Identifying factors that are important and contribute to changes in empathy during educational programs can help participants to cultivate empathy in professional settings and improve existing programs for teaching and maintaining empathic behavior.

The theoretical frame of understanding the construct of empathy adopted in this re-search allowed us to look at empathy from the perspective of readiness for an emotional empathic response combined with the ability to intellectually understand the situation of another person [14]. This approach to empathy leads to reflection on the potential role of various variables that can be damaging or supportive of empathic processes. Empathic behavior as a kind of compromise between state and trait components opens the possibil-ity of exploring variables affecting the state of a given person (e.g., general condition) as well as more dispositional conditions (e.g., personality traits). In the context of the first group of variables related to empathy, and included in the broadly defined term general condition (both mental and physical), in the literature we find many studies on stress or burnout, for example [15,16]. In the group of other variables associated with more dispositional conditions, narcissistic tendencies [17], for example, are perceived as related to empathy on the one hand, and agreeableness and community orientation on the other [18].

Our research aims to find variables (or its predictors in particular) related to empathy in a group of medical and social students. The dependent variable in this study was empathy. Connections of this variable were hypothesized with fatigue, stress and quality of life (a group of variables often conditioned by the current situation), on the one hand, and the perceived pleasure with helping, social self-esteem and gender, on the other. We also considered the hypothesis of differences in the severity of fatigue between the groups of medical and social science students.

## 2. Materials and Methods

### 2.1. Procedure and Participants

Following approval by the local ethics committee, the study was conducted in 2019 and 2020 prior to the COVID-19 pandemic. Participation in the study was voluntary and anonymous. Students from a university and medical university in the north of Poland were invited to take part in the study via the so-called pencil and paper method, and a total of 1701 students from the two universities agreed to participate. The socio-demographic characteristics of the group are presented in Table 1. 

A large majority of the students in the study were women (N = 1366, 81%); male participants comprised 19% (N = 319) of the group. Almost half of the respondents were aged between 21 and 23 (N = 825, 49%), while 37% (N = 634) were aged between 18 and 20, and the remainder were aged 24 and over (N = 236, 14%).

In the medical university, most of the students were studying medicine (N = 414, 41%), nursing (N = 249, 25%), physiotherapy (N = 190, 19%), or midwifery (N = 148, 15%), while most students in the non-medical university were studying psychology (N = 326, 47%), pedagogy (N = 234, 33%), or sociology (N = 140, 20%). The representation of first-year students was slightly higher in the study, which reflects a natural tendency observed at universities. Most students were studying full-time (N = 1665, 98%), but almost half of the surveyed group combined these studies with additional work. 

### 2.2. Methods

The research tool consisted of a short questionnaire to collect basic information about the respondents (age, gender, place of residence) and the university (place, the field of study, year of study), as well as three additional questions related to social self-esteem, prosocial attitude (pleasure of helping others), and subjective quality of life:

(1) “How do you assess the value of yourself?” with possible answers from 0 (“I am not important, others are more important than me”) to 10 (“I am more important than others);
(2) “If you help someone selflessly, how much pleasure does it generally give you?” from 0 (no pleasure at all) to 10 (enormous pleasure);
(3) “How do you estimate the quality of your life?” from 0 (“I am very dissatisfied with my life”) to 10 (“I am very satisfied with my life”).

Empathy was measured using a short, 40-question version of a questionnaire known as the Empathy Quotient [19]. The questionnaire was adapted to Polish conditions, allowing individual differences in empathizing to be measured [20]. The Polish version proved to be comparable to original version regarding psychometric properties of the questionnaire (validitiy and reliability).

To measure the mental and physical aspects of fatigue, an 11-item tool known as the Chalder Fatigue Inventory was used [21]. The Polish adaptation of the test proved to be as reliable and valid as the original tool [22]. 

The PSS-10 (Perceived Stress Scale) is a 10-item tool that globally measures levels of perceived stress experienced during the last month [23]. It has been adapted for scientific purposes (with verified and confirmed reliability and validity) and used widely in Poland in the fields of scientific research and psychological diagnosis. 

All statistical analyses were performed using the Statistica 12 package (StatSoft Inc., Gdansk, Poland). Parametric tests were used in the study. A linear regression analysis was performed and Student’s *t*-test was used to make inter-group comparisons.

## 3. Results

A regression analysis (multiple regression type) was performed to establish which variables determine empathy. The regression model for empathy is presented in Table 2. The results showed that fatigue (β = −0.06, *p* < 0.01), subjectively estimated life satisfaction (β = 0.14, *p* < 0.001), social self-esteem (β = −0.09, *p* < 0.001), the pleasure derived from helping other people (β = 0.29, *p* < 0.001), and gender (β = 0.17, *p* < 0.001) were significant predictors of empathy. The level of stress was the only non-significant predictor of empathy. The whole model is statistically significant (F(6,1665) = 62.28, *p* < 0.05), which allows for the 18% variance of empathy (adjusted R^2^ = 0.1803). Thus, a high quality of life and having feelings of pleasure when helping other people allows us to predict a high level of empathy, especially among females. On the other hand, higher levels of fatigue and social self-esteem, the latter of which is measured here by the belief that you are more important than others, predicts lower empathy.

The next step in the research plan was to compare the students of the two universities in terms of the psychological variables that were significant predictors of empathy. The achieved results are presented in Table 3.

**Figure 1:**
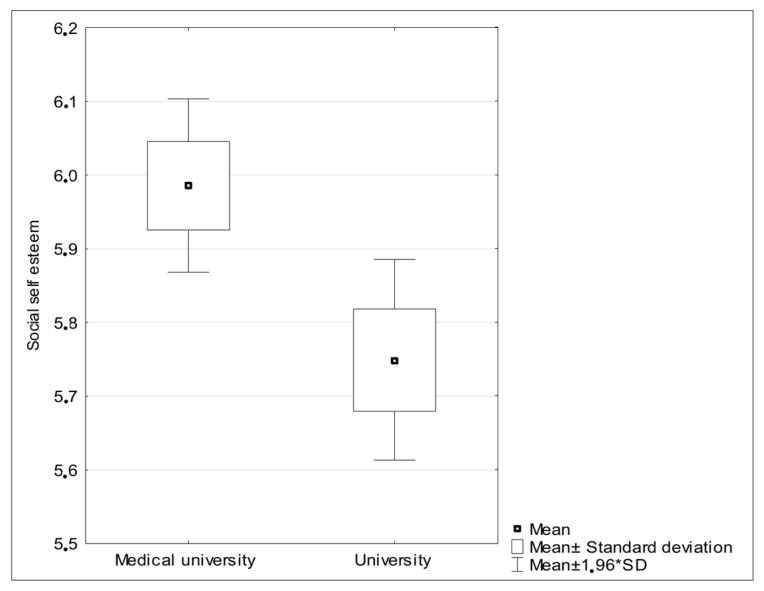
Social self-esteem (comparison of my importance with the importance of others) measured in the group of medical and social science students.

**Figure 2:**
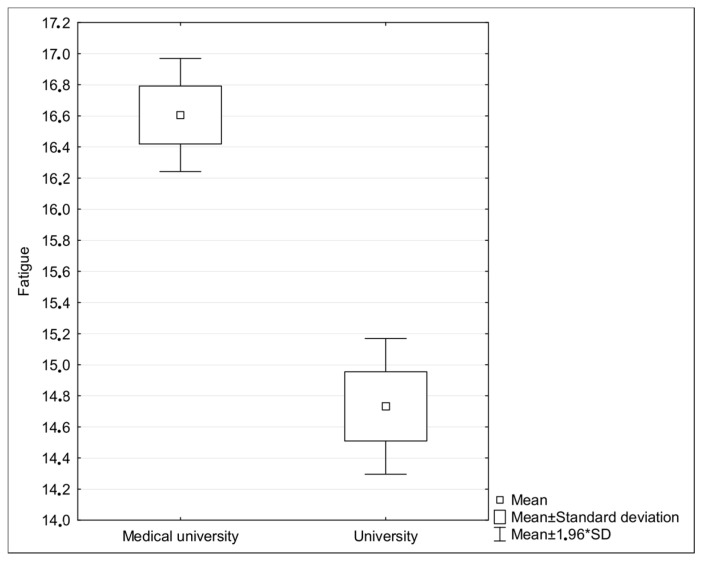
Fatigue (Chalder Fatigue Inventory) in medical students and social science students.

There were no statistically significant differences between students from the two universities when taking into consideration stress levels, subjective quality of life estimations, and prosocial attitude. There were significant differences between fatigue and social self-esteem, however; medical students were both more exhausted (Figure 1) and more convinced of their greater value than other people (Figure 2).

## 4. Discussion

The search for variables related to empathy is important from the perspective of a deep and full understanding of empathy. In our research, almost all assumed variables became important predictors of empathy.

One of the factors significantly related to empathy was undoubtedly gender, a finding that has been well documented in the literature on the subject. In our research, the female gender allowed us to predict stronger empathy, which is consistent with previously described results. Female students show higher empathy scores than male students, even when researchers use different questionnaires to measure empathy [24,25,26,27]. These results are often explained by different cultural and social expectations of gender roles among males and females. Masculine individuals often present higher levels of psychopathy (especially in the areas of boldness and meanness), while feminine individuals typically demonstrate more empathy and cooperation [28,29].

Another statistically significant predictor of empathy was quality of life. Although this area is not as thoroughly explored as the relationships between gender and empathy, we can still find some studies that found similar results [30]. By understanding human behavior not only as a result of personal disposition (i.e., some people are more empathetic than others), but also as an interaction between personal and situational factors, we can attempt to explain the connection between well-being and empathy. Therefore, it is possible that a low quality of life might hinder students’ ability to pay attention to the needs of others; being aware of their own suffering makes it more difficult to notice that someone else needs help [31]. Moreover, maintaining the well-being of workers in helping professions is an even more important issue because it directly influences the quality of their psychosocial care. Preventing work burnout, secondary trauma, and post-traumatic stress disorder and demonstrating the ability to deal with the everyday emotional demands of work influences individuals’ quality of life [32] and helps them to be more empathetic.

Of course, some professional domains of quality of life such as compassion satisfaction is positively associated with dispositional empathy [8]. Our results also indicate that the amount of pleasure one feels in helping others allows us to predict the intensity of empathy, even during studies. A positive feeling associated with helping, which is often connected with patients’ gratitude, and feeling a sense of satisfaction with the work are directly linked to empathy [33].

Because compassion and empathy are so closely related, there is an immense need to maintain a balance between feeling a sense of compassion by caring for others and having self-compassion (e.g., caring for yourself and being kind to self) [34]. Disrupting this balance, especially when it is caused by being too self-judgmental, may lead to a reduced sense of well-being and greater fatigue among students [35].

The ways in which fatigue influences empathy tends to form a vicious circle often described as “compassion fatigue” or even “empathy fatigue.” In our research, fatigue was yet another statistically significant predictor of empathy: greater feelings of fatigue predict a lower level of empathy. This is easily exemplified by people who have dealt with chronic illnesses, disabilities, trauma, death, or grief and are often understood as wounded healers, experiencing exhaustion psychologically, emotionally, mentally, physically, spiritually, and/or occupationally [36]. Compassion fatigue is a form of disengagement that relates to difficulty or inability to empathize with patients. What is interesting, however, is that empathy itself does not lead to compassion fatigue; rather, the main causes include: unbalanced or poor resources (conditional, personal, energetic), a lack of positive feedback (i.e., not being acknowledged, rewarded, or praised), and not having optimal strategies of coping with stress [37]. Thus, people who are already working are much more likely to develop compassion fatigue. In students, a common reason for fatigue might simply be an excessive academic workload, which is responsible for the reduction of empathy; this is especially common among medical students [12]. There is evidences that combining work with studies might also be associated with increased risk of burnout [38], although the latest findings prove that issue of assessing burnout among working students is much more complicated (with the need to take into consideration also gender as key factor) [39]. In our research, the more fatigued the students felt (regardless of the reasons for this fatigue), the less empathy they revealed; we also found that students from the medical university were more exhausted, which indicates the statistically significant difference in the intensity of fatigue.

Students studying at the medical university were also more convinced that their value was greater than others, as compared with the students studying social sciences. Medical students might become more cynical as they progress through medical school [40]. In a later study, the same author found that the third year is particularly crucial, as that is when empathy levels significantly decline, remaining low until graduation [41]. According to our results, social self-esteem was a significant predictor of empathy, which indicates that those who think of themselves as better than others are likely to be less empathetic.

Potential limitations in this study might be connected with the predominance of women in the study group, as well as the unequal numbers of students in particular faculties, which reflect the natural proportions of Polish institutions. Additionally, it should be taken into account that the phenomenon of empathy and empathetic communication may have cultural ties; however, the empathy measurement tool we used has been applied to research all over the world in different cultural contexts, which allows for possible benchmarking and comparisons to be made. This study, having a cross sectional design and using self-report measures also faces all the limitations associated with this type of work. In the future, we plan to conduct a longitudinal and experimental study to more fully analyze the links between empathy and other variables.

## 5. Conclusions

Teaching empathic behavior is an extremely important part of education at universities, in order to prepare people to help others as a part of their duties in their careers. As indicated by our study, it seems that measures should also be taken during this process to ensure students’ well-being and reduce their levels of fatigue while also teaching them how to achieve success in education in the future. Learning to balance compassion, a sense of willingness to help, and self-compassion also seems to be important.

## Figures and Tables

**Table 1:** Socio-demographic characteristics of the participants.

	Students (N; %)
Age (in years)	
18–20	634 (37%)
21–23	825 (49%)
24 and above	236 (14%)
Gender (N; %)	
Female	1366 (81%)
Male	319 (19%)
Type of University	
Medical university	1001 (59%)
University	700 (41%)
Year of Studies	
First-year	472 (28%)
Second-year	323 (19%)
Third-year	292 (17%)
Fourth-year	311 (18%)
Fifth-year	297 (18%)
Type of Studies	
Full-time studies	1665 (98%)
Extramural studies	34 (2%)
Combining Studies with Work	
Only studying	869 (51%)
Additional work during studies	820 (49%)

**Table 2:** Regression model of empathy with predictors for students.

Coefficient	Estimate β	*t*	*p*
Stress (PSS-10)	−0.01	−0.37	0.71
Fatigue (CHFQ)	−0.06	−2.34	0.01
Quality of life	0.14	4.96	0.000001
Social self-esteem	−0.09	−3.79	0.0001
The pleasure of helping others	0.29	12.5	0.0000001
Gender	0.17	7.64	0.0000001

**Table 3:** Comparisons between medical and social science students (Student’s *t*-test).

	Medical University Students (N = 1001) M (SD)	University Students (N = 700) M (SD)	*t*	*p*
Stress	20.17 (10.32)	19.64 (7.27)	1.20	0.23
Fatigue	16.61 (5.98)	14.73 (5.93)	6.45	0.0000001
Quality of life	6.71 (2.00)	6.53 (2.24)	1.75	0.07
Social self-esteem	5.98 (1.94)	5.75 (1.88)	2.57	0.01
Pleasure of helping others	8.39 (1.48)	8.33 (1.51)	0.76	0.44

## Data Availability

Not applicable.
